# Multisensor-Based Target-Tracking Algorithm with Out-of-Sequence-Measurements in Cluttered Environments

**DOI:** 10.3390/s18114043

**Published:** 2018-11-20

**Authors:** Ihsan Ullah, Muhammad Bilal Qureshi, Uzair Khan, Sufyan Ali Memon, Yifang Shi, Dongliang Peng

**Affiliations:** 1 Department of Electrical Engineering, COMSATS University, Abbottabad Campus, Abbottabad 22060, Pakistan; ihsan@ciit.net.pk (I.U.); Bilalqureshi@ciit.net.pk (M.B.Q.); Uzairkhan@ciit.net.pk (U.K.); 2 Department of Electrical and Computer Engineering, Ulsan National Institute of Science and Technology, 50, UNIST-gil, Ulsan 44919, Korea; Sufyanahmedali@gmail.com; 3 School of Automation, Hangzhou Dianzi University, Xiasha Higher Education Zone, 2rd Street, Hangzhou 310018, China; dlpeng@hdu.edu.cn

**Keywords:** information fusion, Kalman filter, out-of-sequence measurements, Rauch–Tung–Striebel, smoothing, state estimation, Time to Impact

## Abstract

A localization and tracking algorithm for an early-warning tracking system based on the information fusion of Infrared (IR) sensor and Laser Detection and Ranging (LADAR) is proposed. The proposed Kalman filter scheme incorporates Out-of-Sequence Measurements (OOSMs) to address long-range, high-speed incoming targets to be tracked by networked Remote Observation Sites (ROS) in cluttered environments. The Rauch–Tung–Striebel (RTS) fixed lag smoothing algorithm is employed in the proposed technique to further improve tracking accuracy, which, in turn, is used for target profiling and efficient filter initialization at the targeted platform. This efficient initialization increases the probability of target engagement by increasing the distance at which it can be effectively engaged. The increased target engagement range also reduces risk of any damage from debris of the engaged target. Performance of the proposed target localization algorithm with OOSM and RTS smoothing is evaluated in terms of root mean square error (RMSE) for both position and velocity, which accurately depicts the improved performance of the proposed algorithm in comparison with existing retrodiction-based OOSM filtering algorithms. The effects of assisted target state initialization at the targeted platform are also evaluated in terms of Time to Impact (TTI) and true track retention, which also depict the advantage of the proposed strategy.

## 1. Introduction

Detection and tracking antiship supersonic targets, such as sea-skimming missiles, are challenging problems for any countermeasure and surveillance system [1]. Various detection sensors, such as RADAR, LADAR, SONAR, and infrared, can be deployed for counteractions to defend against high-speed incoming missiles. Multisensor data fusion is a promising approach to combine the data of multiple sensors having similar or complementary characteristics [2]. Considerable research and development have been carried out towards algorithm development for target dynamics estimation based on information fusion from multiple sensor platforms [2,3,4,5]. Data fusion not only proves useful in terms of estimation accuracy but also reduces susceptibility to Electronic Counter Measures (ECM) by utilizing the benefits of different measurement principles. Despite the numerous advantages of multiple sensor-tracking systems, many practical problems arise in the actual deployment of automated multiple-sensor systems. The issues that must be addressed in data-fusion techniques include dissimilar measurement acquisition rates, resolution capability, and measurement accuracy of the sensors [6].

Measurement acquisition rates differ widely depending on several variables. Therefore, when fusing data from multiple sensors, there is a mismatch in measurement arrival time; for example, data processed from an image processor will be more time-consuming compared to a radar signal being processed. Measurement delays occur due to sensor diversity, transmission delay in the communication network, and uncertainty in preprocessing times when large numbers of measurements are processed in limited resources [6]. As a result, measurements from the same target appear at the fusion center with varying random time delays, and measurements of such a kind are called out-of-sequence measurements (OOSMs). In the OOSM scenario, the fusion center receives measurements produced at a prior time. As explained earlier, this may occur if the measurement arrival was subject to unexpected transmission delays when compared to the delays associated with the expected measurement arrival time. The issue here is that of determining a process of including these OOSMs into a track that has already been updated with a measurement at a later time. In recent years, many optimal and suboptimal filtering techniques have been developed for the handling of single and multiple lag OOSMs [7,8]. An important characteristic of all these OOSM filtering techniques is to compute state estimates, correlative covariance and cross-covariance within the state and delayed measurement [7].

Numerous OOSM filtering techniques have been proposed in the past to incorporate arbitrarily delayed target measurements in an effective manner. Although effective, most of these algorithms do not consider a cluttered environment. This is mainly because of the complexity that accompanies data-association techniques. An approach that takes advantage of the information filter recursions for the OOSM update is proposed in Reference [9], which is a compromise between memory requirement and estimation accuracy. OOSM filtering techniques based on EKF for nonlinear systems to deal with multiple lag OOSMs are proposed in References [10,11]. The particle filter (PF) technique determining the exact solution for the OOSM problem is developed in Reference [12]; however, the technique is numerically very complex. A PF-based solution to the OOSMs with arbitrary lag is presented in Reference [13] for scenarios with bearings-only tracking. The performance of the proposed technique for mildly nonlinear tracking problems is similar to that of an EKF. The performance of any PF-based approach can be further improved by other techniques developed in the literature [14] at the cost of increased computational complexity. The performance of a PF-based technique may be optimized in the computational sense using a cost-reference particle filter approach [15], which is suitable for implementation with parallel computing devices but would not be suitable for problems with scarce hardware resources. Likewise, the performance of PF- and KF-based OOSM filtering techniques for multiple lag OOSMs are compared in [16]. Numerical analysis shows that the KF-based OOSM filtering technique is optimal, and a similar filtering performance can be achieved as for the KF with in-sequence measurements. Two general techniques to update the current state in a globally optimal and suboptimal manner for solving single- and multiple-lag OOSM problems are developed in Reference [17]. In Reference [18], an algorithm is proposed to estimate target position by incorporating arbitrary lag OOSMs using grey relational analysis, which is a computationally intensive technique and involves more CPU time in case of multilag delayed observations. In Reference [19], an idea is presented to augment the history of states to solve a multilag OOSM problem. The technique described in Reference [19] provides an optimal solution for cases when measurement origin time matches one of the predefined discretized time steps [20]. In Reference [21], an exact solution to the OOSM problem is derived by developing an efficient variant of an augmented state Kalman filter (AS-KF) called Selected AS-KF (SAS-KF). Likewise, in Reference [22], a multirate filter is designed to track maneuvering and nonmaneuvering targets in an environment of OOSM reporting.

OOSM filtering techniques, in most of the cases, are build on retrodiction. This implies that the current track is predicted backwards to the originating time of the OOSM measurement [8,23,24]. An optimal retrodiction-based OOSM filtering algorithm called A1 algorithm is presented in Reference [8]. This technique is optimal and offers an exact solution only for a scenario where the OOSM arrives between the previous two in sequence and consecutive measurements. The suboptimal solution to this particular problem derived in References [6,8] is referred as B1 algorithm. A solution is provided in Reference [23] to the *l*-lag OOSM in the framework of the B1 algorithm, known as the B*l* algorithm. In the B*l* algorithm, associated covariances are stored for the computation of filter gain from all past sampling intervals. In Reference [24] the optimal and suboptimal algorithms called Al1 and Bl1 are established to solve multiple lag OOSMs in one step. The aforementioned OOSM filtering algorithms can incorporate single- as well multiple-lag OOSMs with a considerable reduction in estimation error.

For better accuracy, state estimates can be improved further by employing smoothing techniques. These techniques utilize measurements received at intermediate and current scans to improve state estimates at past scans [25]. In Reference [26], the multilag OOSM filtering technique is presented under the framework of fixed lag smoothing. The technique presented in Reference [26] involves state augmentation to include all states up to the first estimated, which makes it computationally inefficient. To limit the computational complexity, an algorithm is proposed in Reference [20], in which RTS smoother is inherently embedded in Accumulated State Density (ASD) filter. Likewise, in Reference [7], a generalized RTS fixed interval smoothing framework is introduced in the OOSM filtering algorithm to incorporate randomly delayed measurements. Recently, the authors in Reference [27] propose an interesting alternative to conventional RTS-based smoothing techniques, which require the target dynamic model information to be accurately known; the fitting for the smoothing method presented in Reference [27] releases the necessity of exact target dynamics and fits the distant estimates given over discrete time by using a function of continuous time, which is then used to infer the state backward for any time instants within the effective fitting period. The simulation experiments show promising results compared to existing smoothing algorithms.

Any nonpersistent measurement that does not originate from a target is referred to as a clutter. The clutter count and spatial distribution are random entities. A general practice in the target-tracking community, when not dealing with clutter estimation, is to assume a homogeneous clutter density that is a priori known [28]. These clutter measurements complicate the situation by introducing uncertainty about the origin of a specific measurement, i.e., whether it belongs to a certain target or not. This problem is addressed by data-association algorithms. Several data-association algorithms exist for single-target tracking, such as nearest neighbor (NN), global NN, probabilistic data association (PDA), integrated PDA, multiple hypothesis tracking (MHT), and integrated track splitting (ITS) [6,28]. While the PDA, IPDA, MHT, and ITS provide the optimal solution, the NN is the least computationally demanding algorithm. It simply assumes that the closest measurement to the predicted track measurement is the one belonging to the target. Due to this computational efficiency, the current algorithm employs this technique over the optimal but computationally intensive algorithms.

In this paper, existing optimal A*l*1 and suboptimal B*l*1 OOSM filtering algorithms based on retrodiction have been proposed along with the smoothing framework to incorporate single- and multiple-lag OOSMs in a cluttered environment. Unlike conventional algorithms, which need multiple steps to update the estimated state, the smoothing enhancement with OOSMs of arbitrary lags are carried out in a single step. Furthermore, this paper presents the additional benefits of smoothing for the purpose of target profiling from several networked Remote Observation Sites (ROSs) along with launch point estimation of high-velocity projectiles. The estimation accuracy of the proposed scheme is demonstrated using computer simulations. The effectiveness of the proposed scheme is validated in terms of RMSE in comparison with existing A*l*1 and B*l*1 OOSM filtering algorithms. Simulations are also carried out for target-state estimation, time-to-impact calculation, and track-retention statistics by the platform protection system of the targeted platform.

The paper is structured as follows: In Section 2, target localization using LADAR and IR with OOSMs is discussed. In Section 3, the problem is formulated and the measurement model of both sensors is illustrated. In Section 4, Estimation and Nearest Neighbor filter recursion equations are explained. Section 5 briefly describes the OOSM filtering algorithms in a cluttered environment with RTS smoothing enhancement. Section 6 describes the advantage of such a system by simulating the platform protection system’s estimation algorithm with and without assisted initialization. Simulation results are presented in Section 7. Finally, Section 8 concludes the proposed work.

## 2. Target Localization Using LADAR and IR

**Figure 1:**
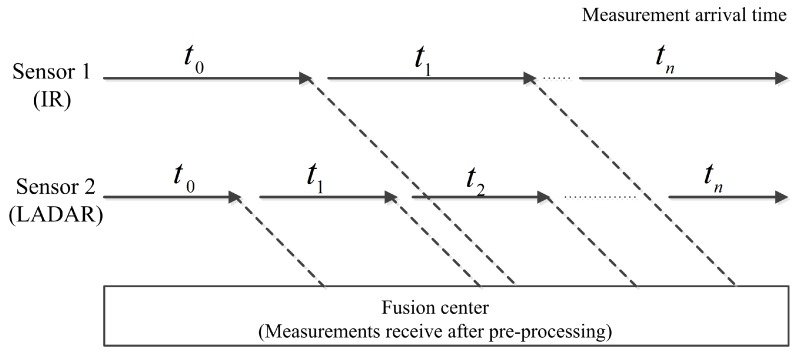
Scenario of out-of-sequence measurements.

This paper considers the information fusion of an LADAR and IR sensor installed on the same platform, separated by a small baseline distance. An assumption is made that both sensors share the same field of view and register the same target in its field of view. The measurements observed from an IR sensor take greater preprocessing time and are not be available at every scan. On the other hand, the measurements from LADAR arrive at the fusion center with a negligible amount of preprocessing time delay as shown in Figure 1. This situation leads to the arrival of IR measurements at the fusion center after the target trajectory state is updated with the measurements observed from LADAR. This creates the problem of including OOSMs into the current state estimate that has already been updated.

**Figure 2:**
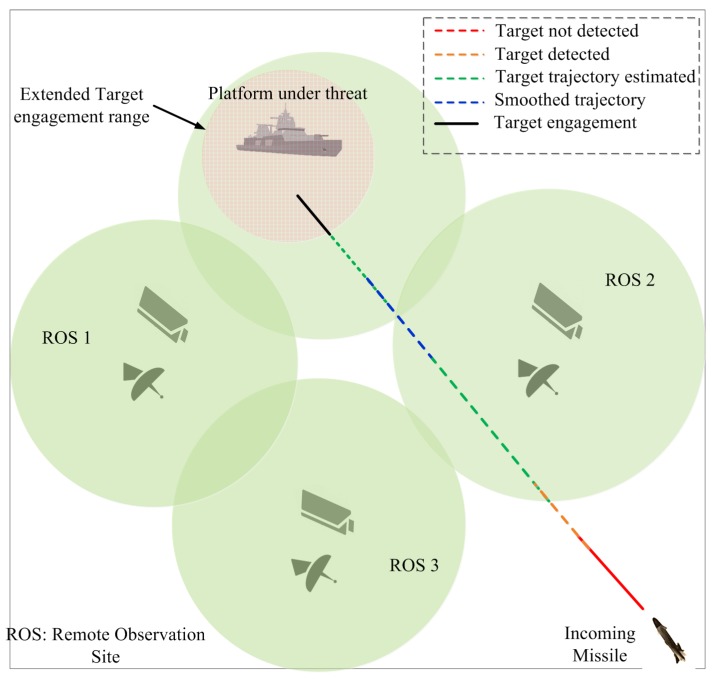
Working of the proposed system with multiple Remote Observation Sites (ROSs) and the targeted platform.

The scenario and use of the algorithm is depicted in Figure 2. A high-speed sea-skimming missile is launched toward the protected platform from a distance. There is always little time between detection and time to impact when tracking a supersonic target in cluttered environment. In the proposed EWS, trajectory of the threat is estimated and smoothed after detection by a ROS. The target trajectory along with accurate time to impact and other parameters of interest are provided in advance to the targeted platform. Thus, the proposed technique helps the ROS in serving as an early-warning system for the targeted platform in practical situations, i.e., tracking with multiple sensors with OOSM and a cluttered environment.

## 3. Problem Formulation

The target state evolves from time $t_{k-1}$ to time $t_{k}$ according to the mathematical expression

(1) $$X_{k}=G_{k,k-1}X_{k-1}+V_{k,k-1}$$

where $G_{k,k-1}$ is the state transition matrix form time $t_{k-1}$ to time $t_{k}$, and $V_{k,k-1}$ is Gaussian-distributed process noise. $X_{k}$ is the state vector that consists of position and velocity components of target in Cartesian co-ordinates. The state vector at any discrete instance *k* can be represented as

(2) $$X_{k}={\left[x_{k}\;\;\;\;y_{k}\;\;\;\;z_{k}\;\;\;\;\dot{x_{k}}\;\;\;\;\dot{y_{k}}\;\;\;\;\dot{z_{k}}\;\right]}^{T}$$

State transition matrix *G* can be defined as

(3) $$G=\left[\begin{array}{c} I_{3\times 3}\;\;\;\;\;\;\;TI_{3\times 3}\\ O_{3\times 3}\;\;\;\;\;\;\;\;\;\;\;\;I_{3\times 3} \end{array}\right]$$

Covariance matrix *Q* that defines process noise $V_{k,k-1}$ can be expressed as

(4) $$Q=q\left[\begin{array}{c} \frac{T^{3}}{3}I_{3\times 3}\;\;\;\;\;\;\;\frac{T^{2}}{2}I_{3\times 3}\\ \frac{T^{2}}{2}I_{3\times 3}\;\;\;\;\;\;\;\;TI_{3\times 3} \end{array}\right]$$

where $I_{3\times 3}$ is a 3×3 identity matrix, $O_{3\times 3}$ is 3×3 zero matrix, *q* is the power spectral density of the process noise, and *T* is the sampling time. It is assumed that the OOSM arrives anywhere in the following interval:

(5) $$t_{k-l}\leq \;\;\tau \;\;<\;\;t_{k-l+1}$$

where τ represents the OOSM arrival time, *k* represents the scan number, and *l* denotes the lag. The measurement equation can be represented as:

(6) $$Z_{s,k}=h^{k}\left(X_{k}\right)+w_{k}$$

where $Z_{s,k}$ denotes the measurement from sensor *s* at time *k*, h is the nonlinear measurement function, and $w_{k}$ is the measurements noise of the sensor. Process noise $V_{k}$ and measurement noise $w_{k}$ are assumed to be mutually uncorrelated, white, zero mean with covariances $Q_{k}$ and $R_{k}$, respectively. According to Equation (1), the state dynamic equation for the OOSM case in can be expressed as:

(7) $$X_{k}=G_{k,\Delta }X_{\Delta }+V_{k,\Delta }$$

where Δ is the discrete time representation of τ. Equation (7) can be rewritten backward as

(8) $$X_{\Delta }=G_{\Delta ,k}\left[X_{k}-V_{k,\Delta }\right]$$

where $G_{\Delta ,k}$ = ${\left(G_{k,\Delta }\right)}^{-1}$ represents the backward transition matrix. The estimated state and covariance matrix can be expressed as:

(9) $$X_{k|k}=E\left[X_{k}|Z^{k}\right],\;\;\;\;\Sigma_{k|k}=\mathrm{cov}\left[X_{k}|Z^{k}\right]$$

where $Z^{k}$ is the set of in-sequence measurements observed from LADAR and is represented as

(10) $$Z^{k}={\left\{z\left(j\right)\right\}}_{j=1}^{k}$$

Subsequently, the OOSM from time τ that can be denoted in discrete time as Δ,

(11) $$z_{\Delta }\;=h_{\Delta }\left(X_{\Delta }\right)+\;w_{\Delta }$$

Measurement $z_{\Delta }$ arrives at the fusion center after state estimate Equation (9) has been calculated. The aim is to update this estimate with delayed measurement Equation (11), by calculating

(12) $$X_{k|\Delta }=E\left[X_{k}|Z^{\Delta }\right],\;\;\;\;\Sigma_{k|\Delta }=\mathrm{cov}\left[X_{k}|Z^{\Delta }\right]$$

where

(13) $$Z^{\Delta }=\left\{Z^{k}\;,z_{\Delta }\right\}$$

### Sensor Measurement Mode

**Figure 3:**
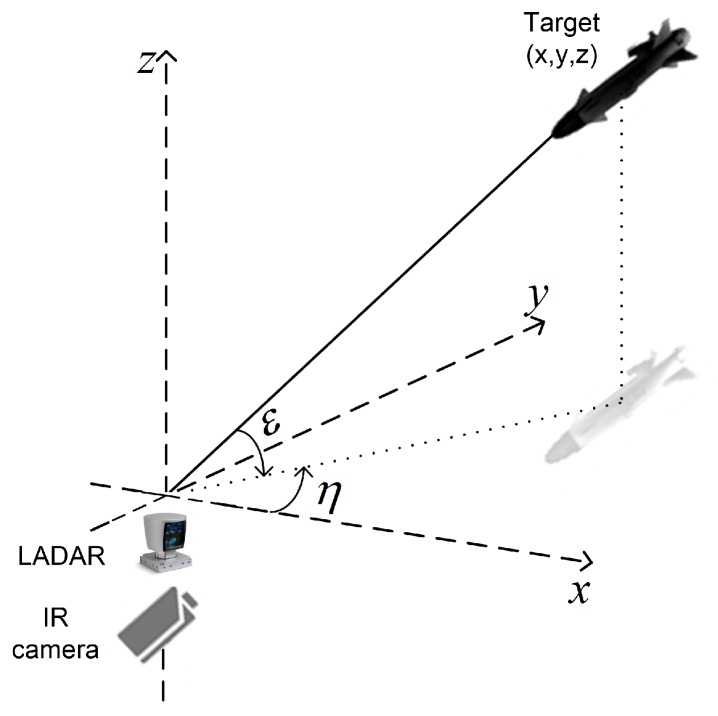
Scenario of out-of-sequence measurements.

The sensor–target geometry in a 3D Cartesian co-ordinate system is clearly depicted in Figure 3. The measurements of LADAR consist of range, azimuth, and elevation angle, whereas the measurement model of IR sensor consists of bearing only measurements, i.e., azimuth and elevation. The LADAR measurements are:

(14) $$r_{k}^{l}=\sqrt{{\left(x_{k}^{t}-x_{k}^{l}\right)}^{2}+{\left(y_{k}^{t}-y_{k}^{l}\right)}^{2}+{\left(z_{k}^{t}-z_{k}^{l}\right)}^{2}}+w_{k}^{l,r}$$

(15) $$\eta_{k}^{l}=\tan^{-1}\frac{y_{k}^{t}-y_{k}^{l}}{x_{k}^{t}-x_{k}^{l}}+w_{k}^{l,\eta }$$

(16) $$\varepsilon_{k}^{l}=\tan^{-1}\frac{z_{k}^{t}-z_{k}^{l}}{\sqrt{{\left(x_{k}^{t}-x_{k}^{l}\right)}^{2}+{\left(y_{k}^{t}-y_{k}^{l}\right)}^{2}+{\left(z_{k}^{t}-z_{k}^{l}\right)}^{2}})}+w_{k}^{l,\varepsilon }$$

where $r_{k}^{l}$, $\eta_{k}^{l}$, $\varepsilon_{k}^{l}$ are the target range, azimuth, elevation angles, respectively. In the same order, the measurement noise for these parameters are represented by $w_{k}^{l,r}$, $w_{k}^{l,\eta }$, and $w_{k}^{l,\varepsilon }$. Superscript *l* here denotes the measurements from LADAR. For the LADAR case, the measurement noise covariance matrix can be expressed as:

(17) $$R_{k}^{l}=diag\left(\sigma_{r_{l}}^{2},\sigma_{\varepsilon_{l}}^{2},\sigma_{\eta_{l}}^{2}\right)$$

IR measurements consist of the target azimuth and elevation angles and can be written as:

(18) $$\eta_{k}^{ir}=\tan^{-1}\frac{y_{k}^{t}-y_{k}^{ir}}{x_{k}^{t}-x_{k}^{ir}}+w_{k}^{ir,\eta }$$

(19) $$\varepsilon_{k}^{ir}=\tan^{-1}\frac{z_{k}^{t}-z_{k}^{ir}}{\sqrt{{\left(x_{k}^{t}-x_{k}^{ir}\right)}^{2}+{\left(y_{k}^{t}-y_{k}^{ir}\right)}^{2}+{\left(z_{k}^{t}-z_{k}^{ir}\right)}^{2}}}+w_{k}^{ir,\varepsilon }$$

Superscript ir represents IR sensor measurements and $\eta_{k}^{ir}$, $\varepsilon_{k}^{ir}$ are the measured azimuth and elevation angles with additive noise represented by $w_{k}^{ir,\varepsilon }$ and $w_{k}^{ir,\varepsilon }$, respectively. The measurement noise covariance matrix of IR is

(20) $$R_{k}^{ir}={\left[\sigma_{\varepsilon_{ir}}^{2},\sigma_{\eta_{ir}}^{2}\right]}^{T}$$

## 4. State Estimation in Cluttered Environments

The Kalman filter is an optimal and recursive algorithm for linear state estimation [29,30]. Prior to fusion, the nonlinear measurements of LADAR are transformed to a Cartesian co-ordinate system, so that linear KF can be employed for state estimation until OOSMs are not received at the fusion center. As indicated from References [31,32,33], converting a nonlinear measurement to the state space yields a non-Gaussian uncertainty and conversion bias; therefore, the critical issue is to determine the unbiased mean and covariance of the observation after conversion. In this paper, we utilize the unbiased conversion approach proposed in Reference [34] to obtain the unbiased mean and covariance of converted measurement in 3D Cartesian space. This debiasing maethod is an exact solution that derives exact compensation for the multiplicative bias and thus shows good consistency and robustness on the statistics of cosine of the angle measurement errors, and is able to apply to non-Gaussian errors as well. For the sake of brevity, the details on the derivation and calculation of the unbiased mean and covariance are omitted here. Interested readers can refer to Section 2B in Reference [34].

The NN filter [6] is employed for target tracking in a cluttered environment. The NN algorithm is based on a few assumptions. The first assumption is that a true target is always detectable; the second is that the measurement nearest to the predicted measurement belongs to the target, and all other measurements originate from the clutter. The third is regarding the white Gaussian nature of the measurement noise. The prediction equations of the linear KF can be mathematically expressed as:

(21) $$X_{k|k-1}=G_{k|k-1}X_{k-1|k-1}+V_{k}$$

(22) $$\Sigma_{k|k-1}=G_{k|k-1}\Sigma_{k-1|k-1}G_{k|k-1}^{T}+Q_{k}$$

The measurement selection process follows these equations, where measurements are selected based on the NN criterion as:

(23) $$z_{k}\left(i\right)=argmin_{z_{k}\left(j\right)}{\left[z_{k}\left(j\right)-H_{k}X_{k|k-1}\right]}^{T}{S_{k}}^{-1}\left[z_{k}\left(j\right)-H_{k}X_{k|k-1}\right]$$

where *i* represents the *i*th track following a target, and $j\in [1,\ldots ,m_{k}]$ and $m_{k}$ represent all the measurements falling in the validation gate, whereas the estimation part, as given in the literature, can be written as:

(24) $$S_{k}=H_{k}\Sigma_{k|k-1}{H_{k}}^{T}+R_{k}$$

(25) $$K_{k}=\Sigma_{k|k-1}{H_{k}}^{T}{S_{k}}^{-1}$$

(26) $$X_{k|k}=X_{k|k-1}+K_{k}\left(z_{k}\left(i\right)-H_{k}X_{k|k-1}\right)$$

(27) $$\Sigma_{k|k}=\left(I-K_{k}H_{k}\right)\Sigma_{k|k-1}$$

where $X_{k|k-1}$ and $\Sigma_{k|k-1}$ are the predicted state estimates and corresponding covariance, $X_{k|k}$ and $\Sigma_{k|k}$ are estimated state vector and covariance, and $S_{k}$, $K_{k}$ represent the innovation covariance and Kalman gain, respectively. Measurement matrix *H* is linear and can be defined as:

(28) $$H_{k}=\left[\begin{array}{c} 1\;\;0\;\;0\;\;0\;\;0\;\;0\\ 0\;\;1\;\;0\;\;0\;\;0\;\;0\\ 0\;\;0\;\;1\;\;0\;\;0\;\;0 \end{array}\right]$$

## 5. OOSM Filtering Algorithms in Clutter With Generalized Smoothing Framework

In this paper, an RTS fixed lag backward smoother is inherently integrated in the framework of existing OOSM and NN algorithms to further improve the estimation and tracking performance. The complete pseudocode of the proposed localization algorithm is shown in Table 1.

### 5.1. Al1 Algorithm with NN and Smoothing Framework

The Al1 algorithm is optimal because it incorporates the retrodicted process noise of the prediction cycle. The algorithm is designed for a multilag OOSM problem but consists of only one step, thus reducing the memory requirement. The key concept behind the approach that can provide a one-step solution to multilag OOSM problems is completely derived in Reference [24] in which all measurements later than OOSM are replaced by an equivalent measurement. The major steps of the algorithm are state retrodiction, measurement retrodiction, and filter-gain calculation that is required to update the current state with OOSM. The retrodiction of the state at *k* to Δ, is given by:

(29) $$X_{\Delta |k}=G_{\Delta ,k}\left[X_{k|k\;\;}-Q_{k,\Delta }{\left(S_{k}^{*}\right)}^{-1}V_{k}^{*}\right]$$

Covariance of equivalent innovation $S_{k}^{*}$ at *k* can be calculated as:

(30) $$S_{k}^{*}=\Sigma_{k|k-l}+R_{k}^{*}$$

The inverse of $S_{k}^{*}$ that is numerically computable is given by:

(31) $${\left(S_{k}^{*}\right)}^{-1}={\left(\Sigma_{k|k-l}\right)}^{-1}-{\left(\Sigma_{k|k-l}\right)}^{-1}{\left[{\left(\Sigma_{k|k-l}\right)}^{-1}+{\left(R_{k}^{*}\right)}^{-1}\right]}^{-1}{\left(\Sigma_{k|k-l}\right)}^{-1}$$

$v_{k}^{*}$ is the equivalent innovation at *k* that can be defined as:

(32) $$v_{k}^{*}={\left(W_{k}^{*}\right)}^{-1}\left[X_{k|k}-X_{k|k-l}\right]$$

where $R_{k}^{*}$ is the measurement noise covariance matrix and $W_{k}^{*}$ is the filter gain for the equivalent measurement, as derived in Reference [23], is:

(33) $${\left(R_{k}^{*}\right)}^{-1}={\left(\Sigma_{k|k}\right)}^{-1}-{\left(\Sigma_{k|k-l}\right)}^{-1}$$

(34) $$W_{k}^{*}=\left(\Sigma_{k|k}\right){\left(R_{k}^{*}\right)}^{-1}$$

The state retrodiction-associated covariances can be calculated as:

(35) $$\Sigma_{vv}\left(k,\Delta |k\right)=Q_{k,\Delta }-Q_{k,\Delta }{\left(S_{k}^{*}\right)}^{-1}Q_{k,\Delta }$$

(36) $$\Sigma_{xv}\left(k,\Delta |k\right)=Q_{k,\Delta }-\Sigma_{k|k-l}{\left(S_{k}^{*}\right)}^{-1}Q_{k,\Delta }$$

where $\Sigma_{vv}\left(k,\Delta |k\right)$ is the process noise covariance associated with state retrodiction, and $\Sigma_{xv}\left(k,\Delta |k\right)$ is the cross-covariance between the state from discrete time Δ*k* to *k*. From Equations (35) and (36), the filter-calculated covariance for a retrodicted state can be calculated as:

(37) $$\Sigma_{\Delta |k}=G_{\Delta ,k}\left[\Sigma_{k|k}+\Sigma_{vv}\left(k,\Delta |k\right)-\Sigma_{xv}\left(k,\Delta |k\right)-{\Sigma_{xv}\left(k,\Delta |k\right)}^{T}\right]G_{\Delta ,k}^{T}$$

Subsequently, the retrodicted OOSM can be defined as:

(38) $$z_{\Delta |k}=H_{\Delta }^{ir}\left(X_{k|k}\right)X_{\Delta |k}$$

The corresponding innovation covariance associated with a retrodicted OOSM is given by:

(39) $$S_{\Delta }=H_{\Delta }^{ir}\left(X_{k|k}\right)\Sigma_{\Delta |k}{\left(H_{\Delta }^{ir}\left(X_{k|k}\right)\right)}^{T}+R_{\Delta }^{ir}$$

The covariance between the current state at time *k* and the retrodicted OOSM can be expressed as:

(40) $$\Sigma_{xz}\left(k,\Delta |k\right)=\left[\Sigma_{k|k}-\Sigma_{xv}\left(k,\Delta |k\right)\right]G_{\Delta ,k}^{T}{\left(H_{\Delta }^{ir}\left(X_{k|k}\right)\right)}^{T}$$

The state can be updated by computing gain $W_{\Delta ,k}$ by the equation

(41) $$W_{\Delta ,k}=\Sigma_{xz}\left(k,\Delta |k\right){\left(S_{\Delta }\right)}^{-1}$$

Current state estimate $X_{k|\Delta }$ can be updated using the OOSM $z_{\Delta }$ by the mathematical equation given as:

(42) $$X_{k|\Delta }=X_{k|k}+W_{\Delta ,k}\left[z_{\Delta }-z_{\Delta |k}\right]$$

The covariance associated with updated state $X_{k|\Delta }$ can be calculated as:

(43) $$\Sigma_{k|\Delta }=\Sigma_{k|k}-\Sigma_{xz}\left(k,\Delta |k\right){\left(S_{\Delta }\right)}^{-1}{\Sigma_{xz}\left(k,\Delta |k\right)}^{T}$$

Measurement matrix $H_{\Delta }^{ir}\left(X_{k|k}\right)$ is nonlinear due to IR nonlinear measurements, and can be expressed as a Jacobian matrix that can be defined as:

(44) $$H_{\Delta }^{ir}\left(X_{k|k}\right)=\left[\begin{array}{c} \frac{\partial \varepsilon_{k}^{ir}}{\partial x}\;\;\frac{\partial \varepsilon_{k}^{ir}}{\partial y}\;\;\;\;\frac{\partial \varepsilon_{k}^{ir}}{\partial z}\;\;0\;\;0\;\;0\\ \frac{\partial \eta_{k}^{ir}}{\partial x}\;\;\frac{\partial \eta_{k}^{ir}}{\partial y}\;\;\;\;\;\;\;0\;\;\;\;\;\;\;\;0\;\;0\;\;0 \end{array}\right]$$

Backward state transition matrix $G_{\Delta ,k}$ can be defined as:

(45) $$G=\left[\begin{array}{c} I_{3}\;\;\;\;\;\;\;\left(-Tl\right)I_{3}\\ O_{3}\;\;\;\;\;\;\;\;\;\;\;\;I_{3} \end{array}\right]$$

Fused estimates $X_{k|\Delta }$ and covariances $\Sigma_{k|\Delta }$, calculated by Equations (42) and (43), are applied to the RTS backward recursions that are embedded in the framework of the A*l*1 algorithm. The RTS backward recursions can be mathematically expressed as:

(46) $$G_{k}=\Sigma_{k|\Delta }\;G_{k}^{T}\;\Sigma_{k+1|\Delta }^{-1}$$

(47) $$X_{k|k}=X_{k|\Delta }+G_{k}\left(X_{k+1|k}-X_{k+1|\Delta }\right),\;\;k=M-1,.....,0$$

(48) $$\Sigma_{k|k}=\Sigma_{k|\Delta }+G_{k}\left(\Sigma_{k+1|k}-\Sigma_{k+1|\Delta }\right)G_{k}^{T}$$

where $G_{k}$ is the smoother gain matrix, $X_{k|k}$ and $\Sigma_{k|k}$ represent the smoothed target state and the covariance estimates of the *k*th time step, respectively. *M* represents the final time step.

The NN algorithm is incorporated in this scenario since the environment is assumed to be cluttered in the IR case, too. Equation (23) can be modified in this case by replacing $X_{k|k-1}$ by $z_{\Delta |k}$ and $S_{k}^{-1}$ by $S_{\Delta }$, obtained by Equation (39). A number of necessary conditional expressions and constructs are used and incorporated in the NN filter framework for practical reasons.

### 5.2. Bl1 Algorithm with Smoothing Framework

The B*l*1 algorithm is suboptimal because it does not incorporate the retrodicted process noise. The only difference between optimal algorithm A*l*1 and suboptimal algorithm B*l*1,is that the former provides an exact solution, whereas the latter an approximate solution. The retrodiction from the current state at *k* to Δ is given as:

(49) $$X_{\Delta |k}^{B}=G_{\Delta ,k}X_{k|k\;\;}$$

The covariances that are associated with state retrodiction can be expressed as:

(50) $$\Sigma_{vv}^{B}\left(k,\Delta |k\right)=Q_{k,\Delta }$$

where the rest of the equations for the B*l*1 algorithm with a smoothing framework are the same as Equations (36)–(48).

## 6. Assisted State Initialization (ASI)

**Figure 4:**
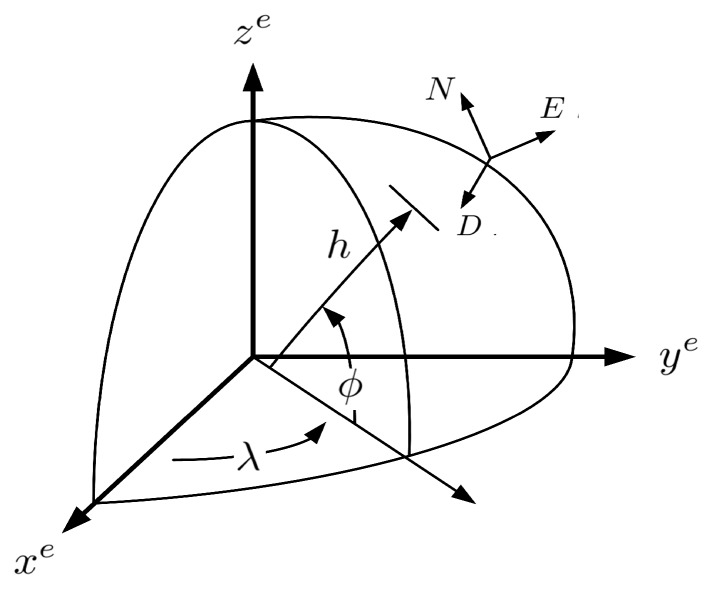
Earth-Centered Earth-Fixed co-ordinate system.

Once the target enters detection range of the platform under threat, the countermeasure system installed in the platform starts estimating the target trajectory for effective engagement. The tracking system of the targeted platform estimates target trajectory in an Earth-Centered Earth-Fixed (ECEF) co-ordinate system with the *z*-axis along Earth’s rotation axis, and the east–north–down co-ordinate system as shown in Figure 4 [35]. This choice of co-ordinate systems is made so that target state estimation at the ROSs can be easily translated for the targeted platform’s tracking system.

Target dynamics are represented is a similar manner as in Section 2. However, in order to measure the performance of the targeted platform’s tracker with and without assisted track initialization, the target state and Time to Impact (TTI) were estimated. Resultant velocity $\dot{R}$ is computed from the estimated state vector and can be mathematically written as:

(51) $$\dot{R}=\sqrt{{\left({\dot{x}}_{k|k}^{P}\right)}^{2}+{\left({\dot{y}}_{k|k}^{P}\right)}^{2}+{\left({\dot{z}}_{k|k}^{P}\right)}^{2}}$$

where ${\dot{x}}_{k|k}^{P}$, ${\dot{y}}_{k|k}^{P}$, and ${\dot{z}}_{k|k}^{P}$ represent the estimated velocity of the target in Cartesian coordinates at the platform. Resultant velocity $\dot{R}$ is chi-distributed with three degrees of freedom and variance, equal to:

(52) $${\sigma^{2}}_{\dot{R}}={\sigma^{2}}_{\dot{x}\dot{y}\dot{z}}\left(3\pi -8\right)/\pi$$

The resultant TTI is also computed and can be mathematically computed as:

(53) $$\mathrm{TTI}=\sqrt{{\left(x_{k|k}^{P}\right)}^{2}+{\left(y_{k|k}^{P}\right)}^{2}+{\left(z_{k|k}^{P}\right)}^{2}}/\dot{R}$$

with $x_{k|k}^{P}$, $y_{k|k}^{P}$, and $z_{k|k}^{P}$ being the estimated target position in Cartesian co-ordinates. The TTI is used to prove the efficacy of the proposed system. The tracking algorithm was the same as in ROSs’ case i.e., NN, whereas statistics of the sensor and surveillance area are given in the Simulation Study section.

## 7. Simulation Study

For both velocity and position, the performance of both the proposed localization and tracking algorithm is compared with the optimal A*l*1 and suboptimal B*l*1 algorithm in a cluttered environment, and is presented in the form of RMSE. The simulation setup considered in this paper is that two sensors (LADAR and IR) are separated by a negligible distance of 0.5 m, and the sensors’ position is defined by position vectors [0,0,10] for LADAR and [0,0,10.5] for IR in the body frame. The target was launched from a distance of 50,000 m, and was moving with constant velocity of 1000 m/s, aimed toward the targeted ship. The ROS estimates the target position for 40 s of its flight. The remaining portion of the trajectory is estimated by the targeted platform with assisted state initialization. The standard deviation of both sensors is defined by $\sigma_{r}^{l}$ = 2.5 m, $\sigma_{\varepsilon }^{l}=\sigma_{\eta }^{l}$ = 12 mrad for LADAR, and $\sigma_{\varepsilon }^{i}r$ = $\sigma_{\eta }^{i}r$ = 5.7 mrad for IR, as given in Reference [1]. The RADAR at the targeted platform has the same parameters as that of the LADAR system.

The measurements from both sensors arrive in such a way that measurements from LADAR are in sequence, whereas a random number of IR measurements are out of sequence with delays of one, three, and five lags. In Table 2, three scenarios are presented. With each measurement, both sensors also provide the time record of the measurement taken. As LADAR measurements are taken at each instant, conventional KF makes the updated state estimate and relevant covariance available. By using the delayed measurements based on optimal or suboptimal OOSM filtering algorithms, the existing target state may possibly be updated. The clutter density for all the sensors, i.e., LADAR, IR, and the RADAR at the targeted platform is ρ=1.0× 10${}^{-5}$/scan/m${}^{2}$, where the clutter follows a uniform distribution and the false measurement count satisfied a Poisson distribution. The probability of target detection is fixed at 0.9 through out the experiment.

**Figure 5:**
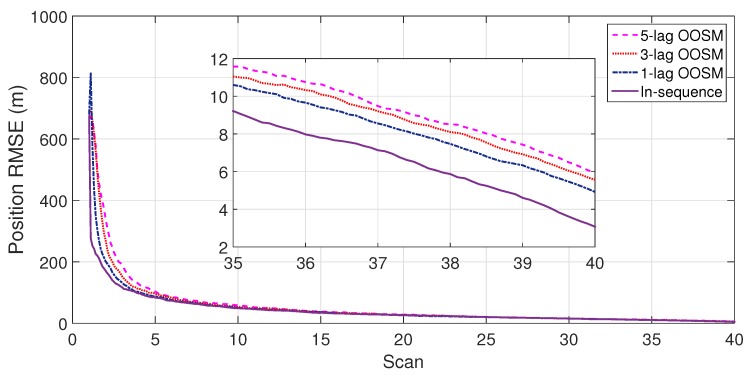
Root mean square error (RMSE) in position estimate for the A*l*1 algorithm.

**Figure 6:**
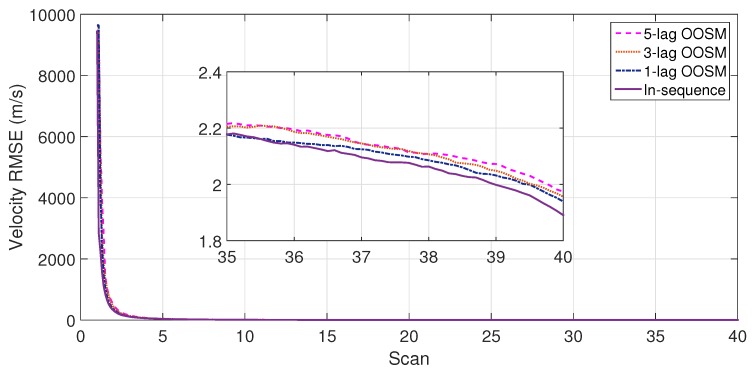
RMSE in velocity estimate for the A*l*1 algorithm.

**Figure 7:**
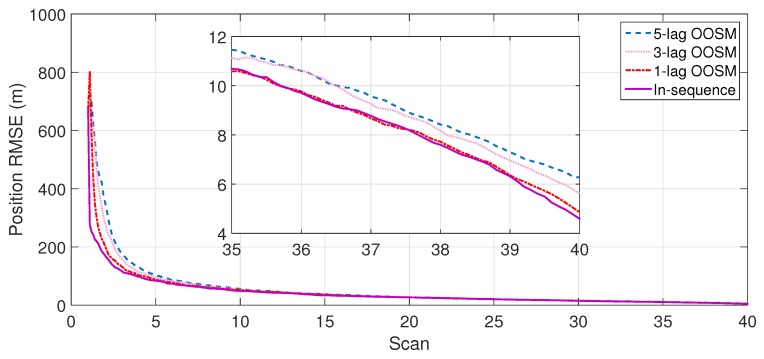
RMSE in position estimate for the B*l*1 algorithm.

**Figure 8:**
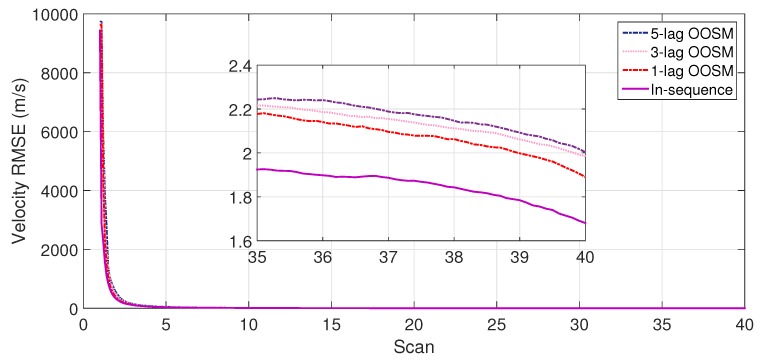
RMSE in velocity estimate for the B*l*1 algorithm.

The experiment consisted of 500 Monte Carlo runs, with each run consisting of 391 scans. For velocity and position, simulation results of the suboptimal and optimal algorithms for both single- and multiple-lag OOSMs in a cluttered environment are presented in Figure 5, Figure 6, Figure 7 and Figure 8 for both position and velocity. The performance of both the OOSM filtering algorithms is also compared to the sequentially obtained readings from IR and LADAR presented in Reference [2]. Based on the results shown in Figure 5, Figure 6, Figure 7 and Figure 8, it is evident that, in terms of RMSE, both algorithms have improved performance estimation. During state retrodiction, the optimal A*l*1 algorithm contains the effects of process noise; however, suboptimal algorithm B*l*1 ignores the process noise during state retrodiction. For multiple-lag OOSMs, such as three and five lags, the RMS error value of both algorithms increases. These RMS error values are presented in Figure 5, Figure 6, Figure 7 and Figure 8. In Table 2 and Table 3, the computational complexity for algorithms A*l*1 and B*l*1 is presented for different lag values and based on the information presented in the table. It is clear that the computational complexity of B*l*1 algorithm is less than that of the A*l*1 algorithm. For multiple-lag OOSMs, computational complexity value increases for both algorithms. From the simulation results, it is observed that performance estimation is identical for both algorithms; however, practical implementations and system requirements are also an important factor for algorithm selection.

In the proposed algorithm, RTS fixed-lag smoothing enhancement is performed in the filtered estimates to further improve the estimation accuracy. In each simulation run, the fixed lag of four and seven scans was considered, and a batch of four and seven scans was formed. The eldest measurement scan was replaced with new measurement scan in each cycle. For a fixed lag value of N = 4, the measurement scans for the first and second batch are j=4,3,2,1, and j=5,4,3,2, respectively, and so on. For fixed value N=7, measurement scans for the first and second batch are j=7,6,5,4,3,2,1, and j=8,7,6,5,4,3,2, and so on.

To limit smoothing delay, an RTS smoothing algorithm that works back in time was implemented in batch form. From the previous examples of fixed lag N=4 and N=7, it is clear that the first output was obtained at j=1 as the algorithm ran for four times for N=4, and seven times for N=7, until the processing of the last batch and achievement of smooth output. At each batch processing, a new smoothed output is obtained. Similarly, the same approach is to be followed for fixed lag N=7. In this case, each time the algorithms run for seven times until the processing of last batch and a smoothed output is obtained.

**Figure 9:**
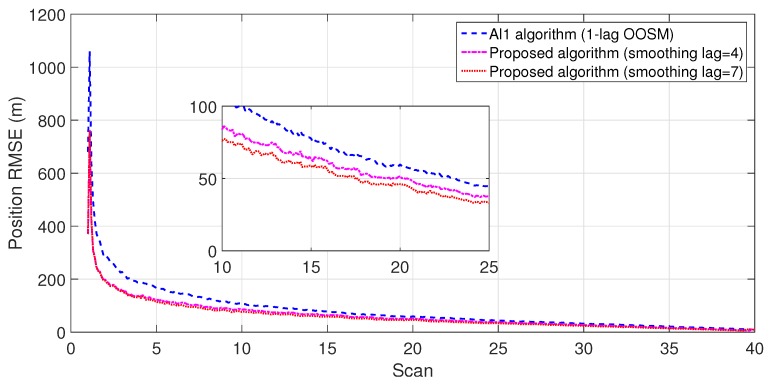
RMSE in position estimate for one-lag OOSM.

**Figure 10:**
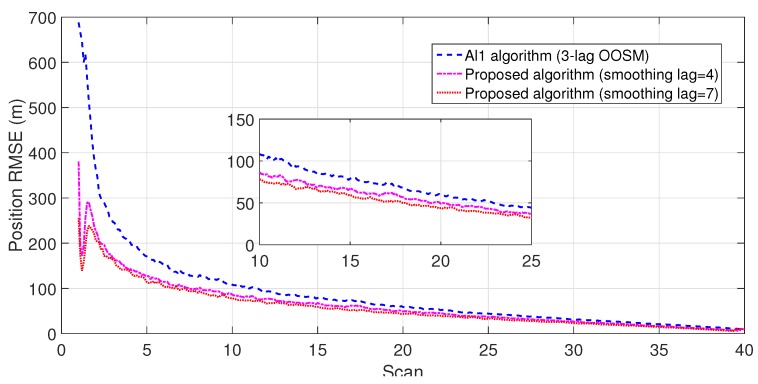
RMSE in position estimate for three-lag OOSM.

**Figure 11:**
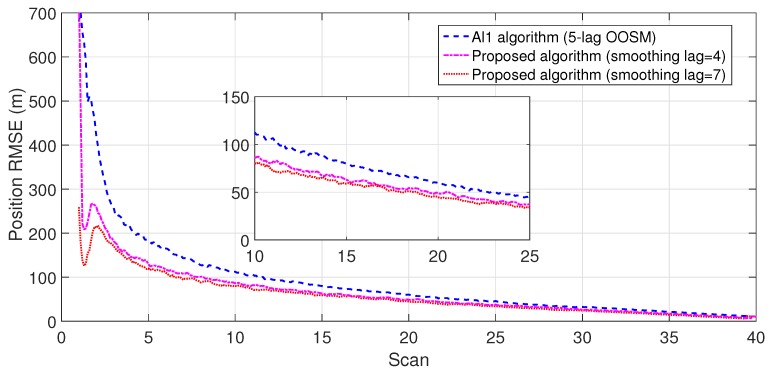
RMSE in position estimate for five-lag OOSM.

**Figure 12:**
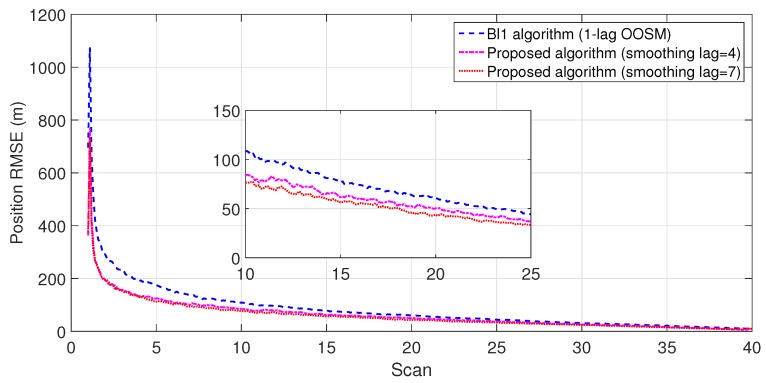
RMSE in position estimate for one-lag OOSM.

**Figure 13:**
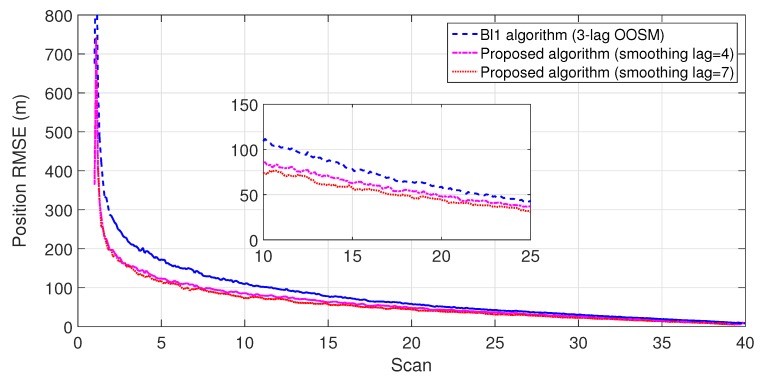
RMSE in position estimate for three-lag OOSM.

**Figure 14:**
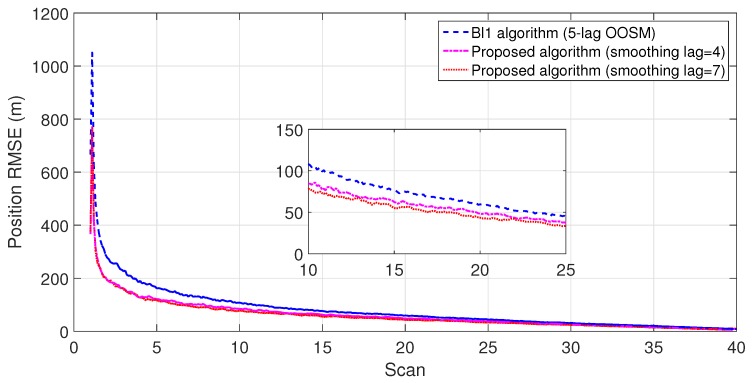
RMSE in position estimate for five-lag OOSM.

**Figure 15:**
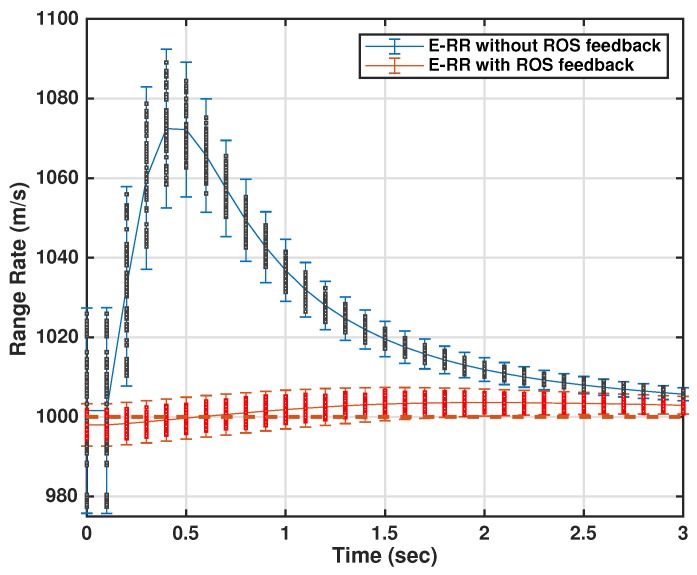
Estimated range-rate at targeted ship’s estimator with and without Assisted State Initialization (ASI).

**Figure 16:**
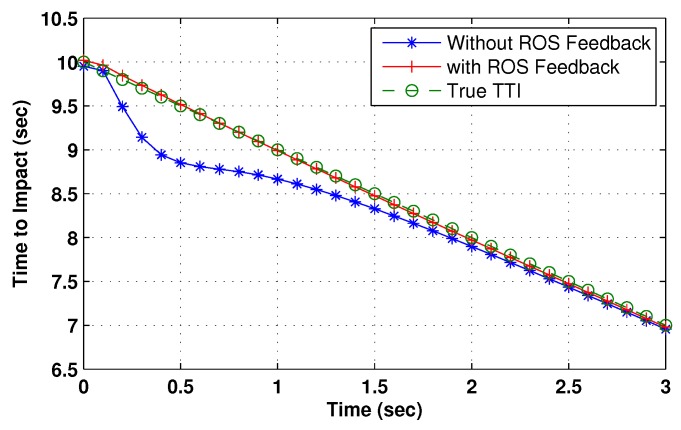
Estimated time to impact at targeted ship’s estimator with and without ASI.

To evaluate the performance of the proposed algorithm with smoothing lag (N=4,7) for one, three, and five lag OOSMs, the experimental statistics were kept as previous. The simulation results related to the comparison of proposed algorithm with Al1 algorithm for position are shown in Figure 9, Figure 10 and Figure 11. The velocity estimation results were omitted since they do not provide a clear picture of the algorithm performance. Similarly, simulation results comparing algorithm Bl1 and the proposed algorithm are displayed in Figure 12, Figure 13 and Figure 14. From the simulation results, the proposed algorithm has been found with better state estimates in the form of reduced RMSE as compared to the existing algorithms. It has also been observed from the simulation results that, as smoothing lag increases, RMSE decreases in terms of velocity and position with increased computational delay. The estimated range rate and TTI with and without ASI are presented in Figure 15 and Figure 16 along with the respective error bounds on range-rate estimate. The results depict the efficacy of the proposed algorithm. It can be observed that there is a 2.5 s advantage to the proposed system when compared with standard state initialization techniques or without ROS feedback. The tracking performance of the filter was also improved with ATI.

The track birth or track-initialization process based on two-point differencing [6] is implemented in the current scenario both at the ROS and the targeted platform tracker. Although single-point track initialization is a much simpler approach since it only requires measurements from the current scan, but the tracks thus formed have no prior information of speed. This lack of information results in a larger gate in the subsequent scan, resulting in a higher number of measurement selections that is not desirable, especially in a scenario with heavy clutter. The two-point differencing approach uses measurements from two consecutive scans for track initialization. This differencing is carried out with all the measurements from the previous scan to the current one; thus, track initialization is based on measurements at the previous scan. A rectangular validation gate that is centered on previous measurements is created with the dimensions of the gate with gating unity probability equal to $2(V_{max}T+2\sqrt{diag(R)})$ [28]. Where $V_{max}$ is the maximum velocity of the target of interest, *T* is the sampling time, and *R* is the measurement covariance matrix.

After the first two scans, any measurement falling in the validation gate of a track is not considered for the track-initialization process. When two or more tracks share a common measurement history for four consecutive scans, these tracks are merged. Similarly, a track is considered lost if the estimation error exceeds five times that of the measurement noise standard deviation.

The simulation in case of the targeted platform tracking system consisted of only 30 scans with the Monte Carlo runs same as in ROS tracking case. A total of five false tracks were reported in both the assisted and nonassisted mode. However, true track retention was much better in the case of ATI, with a total of 50 lost tracks at the final scan as compared to 84 in the case of no feedback from the ROS, which accounts to a 7% improvement in track retention.

## 8. Conclusions

This paper considers the data fusion of a LADAR with IR sensor by incorporating OOSMs to estimate target trajectory and state dynamics of high-speed incoming sea-skimming missiles in a cluttered environment. Optimal and suboptimal OOSM filtering algorithms were incorporated in an NN filter framework to handle single- and multiple-lag OOSMs while tracking the target. A smoothing technique is embedded in the proposed algorithm to further improve the estimation accuracy for assisted track initialization, thus making the tracking system capable enough to deploy countermeasures at an early stage against various high-velocity incoming targets. Monte Carlo simulations clearly demonstrate the improved tracking performance of the proposed algorithm, with a considerable reduction in RMSE and more response time for the countermeasure system as a result. Track retention of the targeted plaform’s tracking system also improved from 83% in the case of missing ROS feedback to 90% in the case with ATI. 

## Figures and Tables

**Table 1:** Typical Out-of-Sequence Measurement (OOSM) scenario for one, three, and five lags.

scene 1	Sensor Number:	1	1	2	1	2	1	2	1	2	1	2	1	2	1
	Measurement arrival time:	0	5	2.5	10	7.5	15	12.5	20	17.5	25	22.5	30	27.5	35
scene 2	Sensor Number:	1	1	1	1	2	1	2	1	2	1	2	1		
	Measurement arrival time:	0	5	10	15	2.5	20	7.5	25	12.5	30	17.5	35		
scene 3	Sensor Number:	1	1	1	1	1	1	2	1	2	1				
	Measurement arrival time:	0	5	10	15	20	25	2.5	30	7.5	35				

**Table 2:** Execution time of the A*l*1 algorithm in a Nearest Neighbor (NN) filter framework for different lags.

Execution Time (s)		
Lag	Scans = 391, Runs = 500	Per Scan
1	33.5	171.3 μ
3	36.2	185.2 μ
5	40.7	208.2 μ

**Table 3:** Execution time of the B*l*1 algorithm in an NN filter framework for different lags.

Execution Time (s)		
Lag	Scans = 391, Runs = 500	Per Scan
1	30.8	157.5 μ
3	34.5	176.5 μ
5	36.1	184.6 μ
